# Insights into thermo-oxidative aging properties and mechanisms of high-content desulfurized rubber modified asphalt: A macro-rheological and micro-chemical perspective

**DOI:** 10.1371/journal.pone.0357088

**Published:** 2026-08-27

**Authors:** Xiaoming Li, Chuanxu Li, Liqiang Xi, Yudong Zhu, Panpan Dong, Peitong Chen, Fan Zhang, Fayong Yang

**Affiliations:** 1 4th Construction Co., Ltd. of China Construction 5th Engineering Bureau, Xi’an, China; 2 Xi’an University of Architecture and Technology, Xi’an, China; 3 Xi’an Highway Research Institute Co., Ltd., Xi’an, China; China Construction Fourth Engineering Division Corp. Ltd, CHINA

## Abstract

To maximize the resource utilization of waste tires, this study systematically investigates the rheological degradation, phase stability, and micro-evolution mechanisms of high-content (30 wt%–50 wt%) desulfurized rubber modified asphalt (HDRA) under short-term and long-term thermo-oxidative aging by rotating film oven test and pressure aging vessel test (RTFOT and PAV). Through multi-scale approaches including DSR, BBR, TD-GC-MS, FTIR, and microscopic morphology characterizations (SEM/FM), the macroscopic rheology, full-lifecycle volatile organic compound (VOC) emissions, and morphological responses were quantitatively evaluated. The results reveal that the 50% HDRA exhibits a unique “aging self-adaptive” characteristic: thermo-oxidative exposure triggers deep swelling and cross-linking of the dense rubber network, which substantially reinforces high-temperature elasticity while restricting particle migration, thereby completely reversing the initial high-temperature segregation susceptibility. However, the hyper-dense skeleton introduces a low-temperature “rheological trade-off”; long-term aging restricts the internal free volume, significantly diminishing the stress relaxation capacity under sub-zero conditions. Furthermore, gaseous molecule tracking and chemical index analysis confirm a microscopic “sacrificial protection” mechanism. The early-stage intensive depolymerization and devulcanization of the rubber network consume thermo-oxidative energy and physically and chemically shield the base asphalt from profound hardening. Consequently, as aging deepens, the full-lifecycle failure mode transitions from conventional matrix embrittlement to severe polymer-asphalt interfacial debonding and phase homogenization. This study provides a novel theoretical foundation for the precise design and eco-friendly application of ultra-high content solid-waste rubber in long-life pavement materials.

## 1. Introduction

With the explosive growth of the global automotive and transportation industries, the generation of waste rubber tires has risen exponentially, rendering the resulting “black pollution” one of the most challenging solid waste management issues worldwide. Processing waste tires into crumb rubber and incorporating it into asphalt is widely recognized as a green, circular pathway that yields simultaneous environmental, economic, and engineering benefits [1–3]. Within the asphalt matrix, rubber particles physically swell by absorbing light fractions and construct an elastic polymer network, thereby significantly enhancing the pavement material’s high-temperature rutting resistance, fatigue life, and elastic recovery [4,5]. However, the rubber dosage in traditional rubber-modified asphalt is strictly limited to 15% to 20% by weight of the base binder. While this lower dosage maintains controllable workability for pavement construction, it generally fails to form a continuous, dominant elastic skeleton throughout the material’s lifecycle [6,7]. Consequently, this limitation severely restricts the long-term durability of traditional rubberized pavements under extreme heavy-load traffic or severe climate conditions.

To maximize the resource utilization efficiency of waste rubber tires and endow paving layers with superior long-term shear resistance, the preparation of high-content rubber modified asphalt (HDRA)—with rubber dosages exceeding 30% or even reaching 50%—has emerged as a prominent research hotspot at the intersection of pavement engineering and polymer blending [8–10]. However, the introduction of ultra-high rubber contents is inevitably accompanied by fatal defects: thermodynamic phase instability and rheological collapse during construction [11]. Due to the crowding effect of high-concentration solid particles and the massive chemical polarity disparity between the rubber polymer and the heterogeneous asphalt matrix, the rubber particles are highly susceptible to severe gravity sedimentation and macroscopic phase segregation during high-temperature storage [12,13]. To overcome this, desulfurized rubber powder (DRP) prepared via polymer degradation and activation mechanisms has been developed. Through the synergistic effects of thermal, mechanical, or chemical agents, the desulfurization process directionally ruptures the high-energy sulfur-sulfur (S-S) and carbon-sulfur (C-S) bonds within the three-dimensional cross-linked network of vulcanized rubber [14–16]. While retaining the elastic carbon backbone, this process significantly enhances the physical shear activity and segment mobility of the rubber surface. This not only profoundly improves the molecular wetting and phase dispersion of ultra-high dosage rubber within the asphalt matrix but also makes the construction of a continuous, interpenetrating composite elastic network feasible.

Nevertheless, throughout the entire service lifecycle, HDRA systems are inevitably subjected to severe thermo-oxidative aging. During the mixing and paving stages (short-term aging) and long-term in-service periods (long-term high-pressure oxidation), the material’s microarchitecture undergoes irreversible physico-chemical cleavage and rheological degradation [17–19]. Compared to conventional low-dosage systems, the aging behavior of HDRA exhibits distinct “non-linear” characteristics and highly complex competitive degradation mechanisms typical of polymer blends. On one hand, the light oil fractions in the asphalt matrix undergo cross-linking and polarity transitions triggered by thermo-oxidation, generating highly polar hydrophilic groups such as carbonyls (C = O) and sulfoxides (S = O), which leads to matrix hardening and embrittlement [20]. On the other hand, the highly concentrated DRP network itself undergoes thermo-oxidative degradation, depolymerization, and secondary radical re-crosslinking [21]. This dynamic, spatiotemporal collision between continuous polymer phase degradation and asphalt matrix hardening dictates the long-term evolution of the material’s high- and low-temperature rheological responses, interfacial adhesion, and multi-phase compatibility [22,23]. Investigating the thermo-oxidative evolution mechanisms of such multi-phase, multi-component polymer blends holds profound academic significance for revealing the fundamental aging failure modes of composite materials from a polymer chemistry perspective.

Furthermore, due to the extreme initial viscosity induced by ultra-high DRP dosages, the processing temperatures for HDRA during preparation, paving, and aging simulations must often be significantly elevated. Under such intense thermal-shear and oxygen-rich environments, the construction and degradation of the continuous polymer network not only involve dynamic internal microstructural adjustments but also inevitably trigger severe secondary emissions of volatile organic compounds (VOCs) and toxic gases [24]. In the modern context of “green and low-carbon” materials and Life Cycle Assessment (LCA), the volatile emission kinetics and the compositional evolution of gaseous small molecules across different aging stages have become core indicators for evaluating the ecotoxicological risks and low-carbon feasibility of HDRA [25,26]. However, existing studies predominantly focus on the fluctuations of macroscopic engineering mechanical indices, often neglecting the dynamic tracking of characteristic gaseous molecules—such as D-Limonene and 4-Vinylcyclohexene (indicators of polymer main-chain thermal depolymerization), Benzothiazole (direct evidence of cross-linked network devulcanization), and environmentally toxic Polycyclic Aromatic Hydrocarbons (PAHs) [27,28]. Without a cross-scale coupled analysis of these gaseous products, it is impossible to fully deconstruct the panorama of “physical sacrifice” and “chemical scission” within the ultra-high content polymer network throughout its lifecycle.

In light of this, this study systematically investigates the macroscopic rheological evolution, phase thermodynamic stability, full-lifecycle volatile emission dynamics, and cross-scale microscopic functional group responses of HDRA under sequential thermo-oxidative aging. Breaking away from a singular engineering evaluation perspective, this research employs a Dynamic Shear Rheometer (DSR) and Bending Beam Rheometer (BBR) to quantitatively assess the macro-micro structural trade-off in high- and low-temperature rheological behaviors. Simultaneously, the Total Volatile Organic Compounds (TVOC) and characteristic gaseous molecules across three representative service stages were analyzed. Furthermore, Scanning Electron Microscopy (SEM), Fluorescence Microscopy (FM), and Fourier Transform Infrared Spectroscopy (FTIR) are combined to quantitatively characterize the micro-phase evolution and polymer oxidation kinetics. This study aims to deconstruct and validate the “aging self-adaptive” mechanism and the “microscopic sacrificial protection” behavior of ultra-high content polymer networks under extreme aging conditions, ultimately providing a novel scientific and theoretical basis for the precise design and eco-friendly application of solid-waste rubber in high-performance, long-life composite materials.

## 2. Materials and methods

### 2.1. Raw materials

#### 2.1.1. Asphalt.

The 90# base asphalt obtained from Sinopec was selected, and its basic indicators are shown in Table 1.

**Table 1:** Basic indicators of 90# BA.

Items		Test value	Requirements
Penetration (25 °C, 100g, 5s) (0.1 mm)		86.3	80 ~ 100
Ductility (5 cm/min, 10 °C) (cm)		79	≥20
Softening point (°C)		45.9	≥45
Viscosity (135 °C) (Pa·s)		0.412	≥0.16
Dynamic viscosity (60 °C) (Pa·s)		228.9	≥160
After TFOT	Mass loss (%)	0.11	±0.8
	Penetration ratio (%)	65.8	≥57
	Ductility (5 cm/min, 10 °C) (cm)	16.4	≥8

#### 2.1.2. Desulfurized rubber powder.

The DRP used in this study was purchased from a manufacturer in Hubei Province, China. This DRP is prepared by deep desulfurization of waste tire rubber powder through a special desulfurization process. DRP was produced via an additive-free, micro-oxygen-assisted thermomechanical process at 200–220 °C under mechanical agitation for 1 h. Under these conditions, the C–S, S–S, and polysulfide crosslinks in CRP are selectively broken, with the released sulfur oxidized into SOx gas and released. Compared with conventional desulfurization methods, this eco-friendly approach prevents severe polymer backbone oxidation without requiring sulfur-removing agents. The basic indicators of DRP are shown in Table 2.

**Table 2:** Basic properties of DRP.

Items	Relative density	Metal content/%	Fiber content/%	Rubber hydrocarbon/%	Ash content/%	Acetone extract/%	Carbon black/%	Mooney viscosity/ ML1 + 4 100°C
Test value	1.21	0.018	0.12	62	5.2	9	34	20

#### 2.1.3. Additives.

Based on preliminary research, a 2 wt% linear styrene-butadiene-styrene (SBS) copolymer and sulfur stabilizer were incorporated as additives. The trace amount of SBS was specifically introduced to establish a baseline thermodynamic storage stability, mitigating the severe premature macroscopic segregation of the ultra-high rubber content during sample preparation and handling prior to rheological evaluations.

### 2.2. Test methods

#### 2.2.1. Preparation of HDRA.

HDRA was prepared utilizing a rigorously controlled high-shear thermo-mechanical blending technique. Initially, the base asphalt was preheated to a fluid state at approximately 150 °C within a customized heating mantle. Subsequently, the 2% SBS and DRP were incrementally incorporated into the molten base asphalt to formulate three distinct dosage levels: 30%, 40%, and 50% by weight of the base asphalt on an add-on basis (external addition, keeping the base asphalt mass constant across all formulations). This approach ensures a consistent bitumen baseline to precisely evaluate the isolated dosage effects of high DRP content.

Given the ultra-high DRP content, the mixture was first stirred for 30 min to facilitate the initial wetting of the rubber particles by the asphalt’s light fractions. Following the pre-mixing phase, the temperature was elevated to 185 °C, and a high-shear mixer was employed to vigorously shear the blend at a constant speed of 4000 rpm for 60 min. Finally, the prepared HDRA blends were stirred for 60 min after adding stabilizer, and then transferred to an oven and allowed to swell statically and mature at 180 °C for 60 min. Following the above steps, HDRA30, HDRA40, and HDRA50 were prepared.

#### 2.2.2. Simulation of thermal-oxidative aging.

(1) Rotating film oven test (RTFOT)

To simulate the short-term thermal-oxidative aging behavior of HDRA during the high-temperature plant mixing and paving process, RTFOT was conducted. According to the standard ASTM D2872 procedure, the RTFOT is typically conducted at 163 °C. Notably, due to the ultra-high viscosity imparted by the super-high DRP content, the standard aging temperature was elevated to 175 °C to ensure proper thin-film formation within the glass bottles. This modification was implemented for two main reasons. Firstly, HDRA exhibits significantly higher viscosity at 163 °C compared to conventional binders, which restricts its flowability and prevents the formation of a uniform thin film inside the RTFOT bottles, potentially leading to an underestimation of the aging extent [29]. Secondly, the RTFOT aims to simulate the short-term aging during plant mixing and field paving. Since the actual production temperature of HDRA is typically elevated to approximately 175 °C–185 °C to ensure workability, an aging temperature of 175 °C provides a more realistic simulation of its actual thermo-oxidative history in the field [30].

Unaged binder was rotated horizontally at 15 rpm for 85 minutes under the elevated temperature, accompanied by a continuous hot air flow of 4000 mL/min. After RTFOT aging, the HDRA30-R, HDRA40-R, and HDRA50-R were obtained.

(2) Pressure aging vessel test (PAV)

To simulate the long-term in-service oxidative aging of the HDRA, the PAV test was conducted in accordance with ASTM D6521. The samples were subjected to an applied air pressure of 2.1 MPa at a temperature of 100 °C for a continuous duration of 20 hours. Notably, due to the extremely high viscosity of the high-content rubberized asphalt, the subsequent vacuum degassing process was performed at an elevated temperature of 170 °C for 30 minutes to ensure the complete removal of entrapped air bubbles before low-temperature rheological evaluations. After PAV aging, the HDRA30-P, HDRA40-P, and HDRA50-P were obtained.

#### 2.2.3. Macroscopic tests.

(1) Basic properties

The basic properties, including penetration, softening point, ductility, and elastic recovery of HDRA, were evaluated according to ASTM D5, ASTM D36, ASTM D113, and ASTM D6084, respectively.

(2) Segregation test

The high-temperature storage stability and phase segregation potential of the HDRA were evaluated using a separation test in accordance with ASTM D7173. The segregation severity was quantitatively characterized by measuring the difference in the ring-and-ball softening points between the top and bottom sections, which directly reflects the macroscopic stability and interfacial compatibility of the polymer-asphalt blend under prolonged thermal exposure.

#### 2.2.4. Rheological properties.

(1) Temperature sweep test

The high-temperature rheological properties and rutting resistance of HDRA were evaluated using a Dynamic Shear Rheometer with test temperatures ranging from 52 °C to 88 °C and a frequency of 10 rad/s. The complex shear modulus (G*) and phase angle (δ) were continuously recorded to determine the rutting factor (G*/sinδ).

(2) Bending beam rheometer test

The low-temperature stress relaxation capacity and cracking susceptibility of HDRA networks were assessed using a Bending Beam Rheometer, with the temperature of −12 °C to −24 °C. The critical low-temperature performance indices: creep stiffness (S) and creep rate (m-value) were obtained.

#### 2.2.5. Asphalt fume and VOCs detection.

To investigate the life-cycle VOCs emission behavior of HDRMA, a dynamic gas collection system was employed across three stages:

Unaged Stage: different HDRAs were heated at 170°C and mechanically stirred for 30 min in a sealed reactor to simulate the thermal-oxidative degradation and secondary emission of the asphalt binder material under extreme thermal processing.

Short-term Aging Stage: Fumes were captured in-situ from the exhaust outlet of the oven throughout the standard RTFOT process to track production plant emissions.

Long-term Aging Stage: Because deeply aged in-service pavements do not release significant gaseous emissions at ambient temperatures, the PAV-aged residues were deliberately reheated to 170 °C and stirred under identical conditions. This protocol strictly simulates the secondary volatile emissions and ecotoxicological risks associated with the hot-in-place recycling operations of severely aged pavements.

For the Unaged Stage and Long-term Aging Stage, air was introduced at a flow rate of 1.0 L/min to sweep volatiles into sorbent tubes. All collected samples were analyzed via Thermal Desorption-Gas Chromatography-Mass Spectrometry (TD-GC-MS) to determine total volatile organic compounds (TVOC) concentrations and chemical compositions.

#### 2.2.6. Microscopic Characterizations.

(1) Scanning electron microscope (SEM)

The microscopic morphology and phase distribution of the DRP within HDRA were characterized using a JSM-7610F Scanning Electron Microscope. To systematically evaluate the global dispersion state, agglomeration behavior, and interfacial compatibility of the varying DRP dosages, the microscopic images were uniformly captured at a magnification of 500 × .

(2) Fluorescence microscope (FM)

The phase distribution, spatial network morphology, and microstructural compatibility between DRP and the base asphalt were visually characterized using a BX53 Fluorescence Microscope. To systematically capture the representative macroscopic dispersion state, the formation of distinct rubber strips, and the subsequent structural fragmentation induced by thermal-oxidative aging, all optical morphological images were uniformly captured and recorded at a magnification of 40 × .

(3) Fourier transform infrared spectroscopy (FTIR)

The functional group variations of the HDRA during thermal-oxidative aging were characterized using Nicolet iS50. To obtain high-quality spectra without complex sample pretreatment, the Attenuated Total Reflectance (ATR) mode was employed. The spectra were recorded in the wavenumber range of 4000−400 cm^-1^ with a resolution of 4 cm^-1^ and 32 cumulative scans per sample.

To further quantitatively assess the aging severity of the HDRA, Structural Indices (SI) based on functional groups were calculated. Given that the aliphatic string vibrations at around 1460 cm^-1^ and 1375 cm^-1^ remain highly stable during the thermal-oxidative process, the peak area surrounding 1460 cm^-1^ was selected as the reference baseline [31]. The Carbonyl Index (I_C=O_), Sulfoxide Index (I_S=O_), and Butadiene Index (I_C=C_) were defined and calculated using the following equations:


$$\begin{array}{c} I_{C=O}=\frac{A_{1700}}{\sum A} \end{array}$$ (1)

$$\begin{array}{c} I_{S=O}=\frac{A_{1030}}{\sum A} \end{array}$$ (2)

$$\begin{array}{c} I_{C=C}=\frac{A_{966}}{\sum A} \end{array}$$ (3)

where *A*_*1700*_, *A*_*1030,*_ and *A*_*966*_ represent the integrated areas of the absorption peaks centered at approximately 1700 cm^-1^ (stretching vibration of C = O), 1030 cm^-1^ (stretching vibration of S = O), and 966 cm^-1^ (C = C bending vibration), respectively. *∑A* represents the reference peak area, calculated as the total integrated area of the C-H bending vibrations located between 1520 cm^-1^ and 1350 cm^-1^.Integrating this broader regional band, rather than relying on isolated singular peaks, effectively minimizes baseline shift errors caused by the severe multi-phase polymer degradation and structural overlapping during advanced aging states.

## 3. Results and discussion

### 3.1. Basic properties analysis

**Fig 1:**
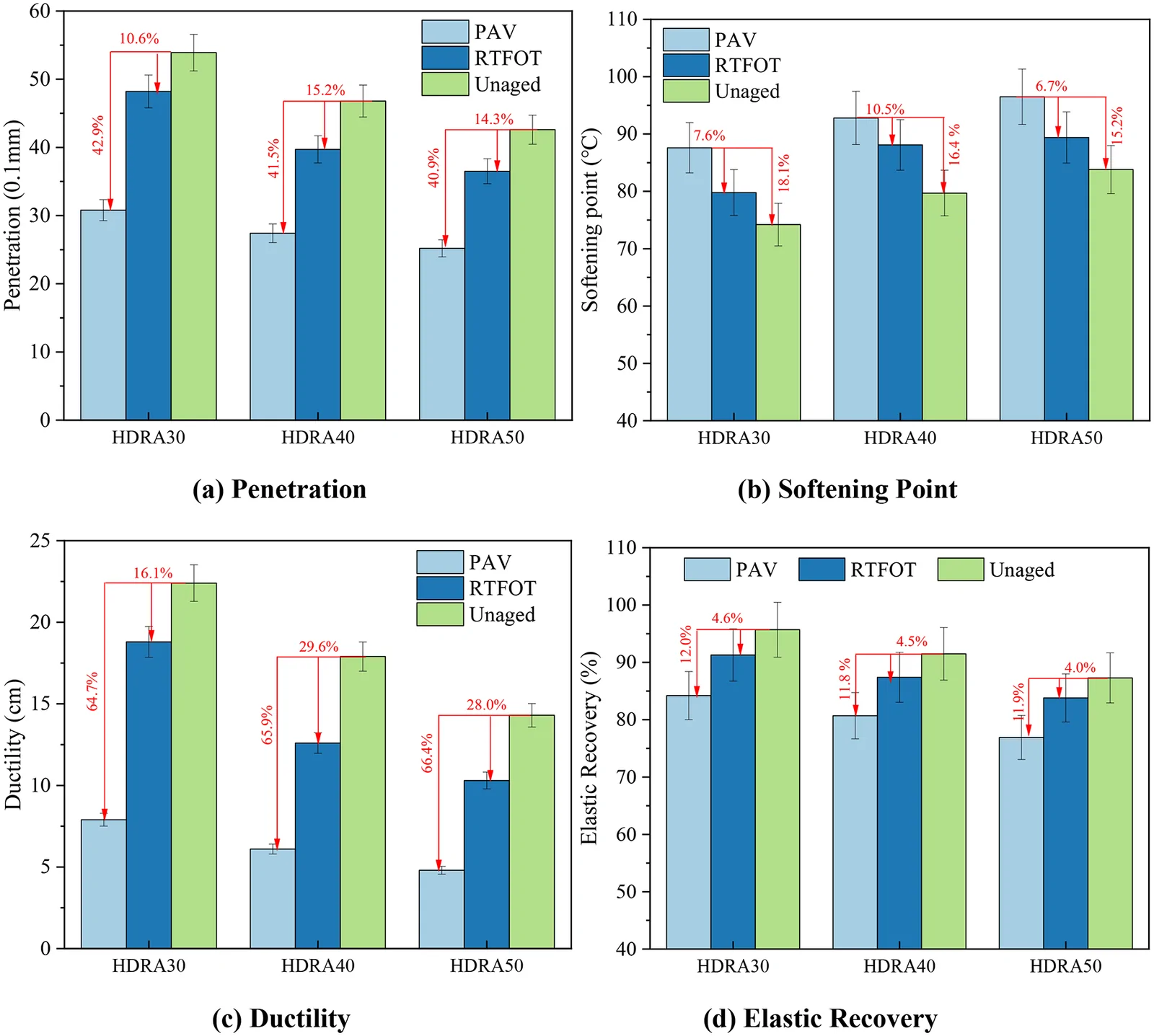
Basic properties of HDRA. (a) Penetration, (b) Softening Point, (c) Ductility, (d) Elastic Recovery.

In the unaged state, increasing DRP from 30% to 50% establishes a highly dense spatial network, empirically evidenced by the initial softening point elevating significantly from 72.9 °C to 81.9 °C. Upon standard thermo-oxidative (RTFOT) and long-term (PAV) aging, the distinct dosage systems display divergent rheological susceptibilities. The HDRA30 experiences characteristic oxidative hardening, with its softening point surging to 87.0 °C post-PAV, primarily driven by the volatilization of light fractions and matrix oxidation. Conversely, HDRA50 demonstrates an exceptional “aging self-adaptive” capability. Despite rigorous PAV conditioning, it maintains profound stability (softening point reaching 95.5 °C) without precipitous embrittlement. This mechanism originates from dynamic phase synergy: the thermo-oxidative environment inevitably stiffens the asphalt matrix, but it concurrently triggers the progressive deep swelling and chemical cross-linking of the densely packed DRP particles. This microscopic evolution dynamically compensates for the matrix hardening, thereby preserving the structural integrity of the three-dimensional elastic network throughout the aging lifecycle (Fig 1) [32,33].

Furthermore, the mechanical response evolution under aging highlights how the high-dosage skeleton fundamentally reshapes the material’s failure mode. While lower-dosage binders typically suffer severe degradation in tensile flexibility post-PAV due to matrix embrittlement, the mechanical feedback of the 50% HDRA system is predominantly governed by its continuous elastic polymer network. Driven by this dual chemical-physical anti-aging mechanism and dense network reconstruction, the 50% HDRA system successfully balances high-temperature deformation resistance with long-term cracking toughness, validating the structural superiority of ultra-high dosage networks against severe oxidative degradation.

### 3.2. Storage stability analysis

**Fig 2:**
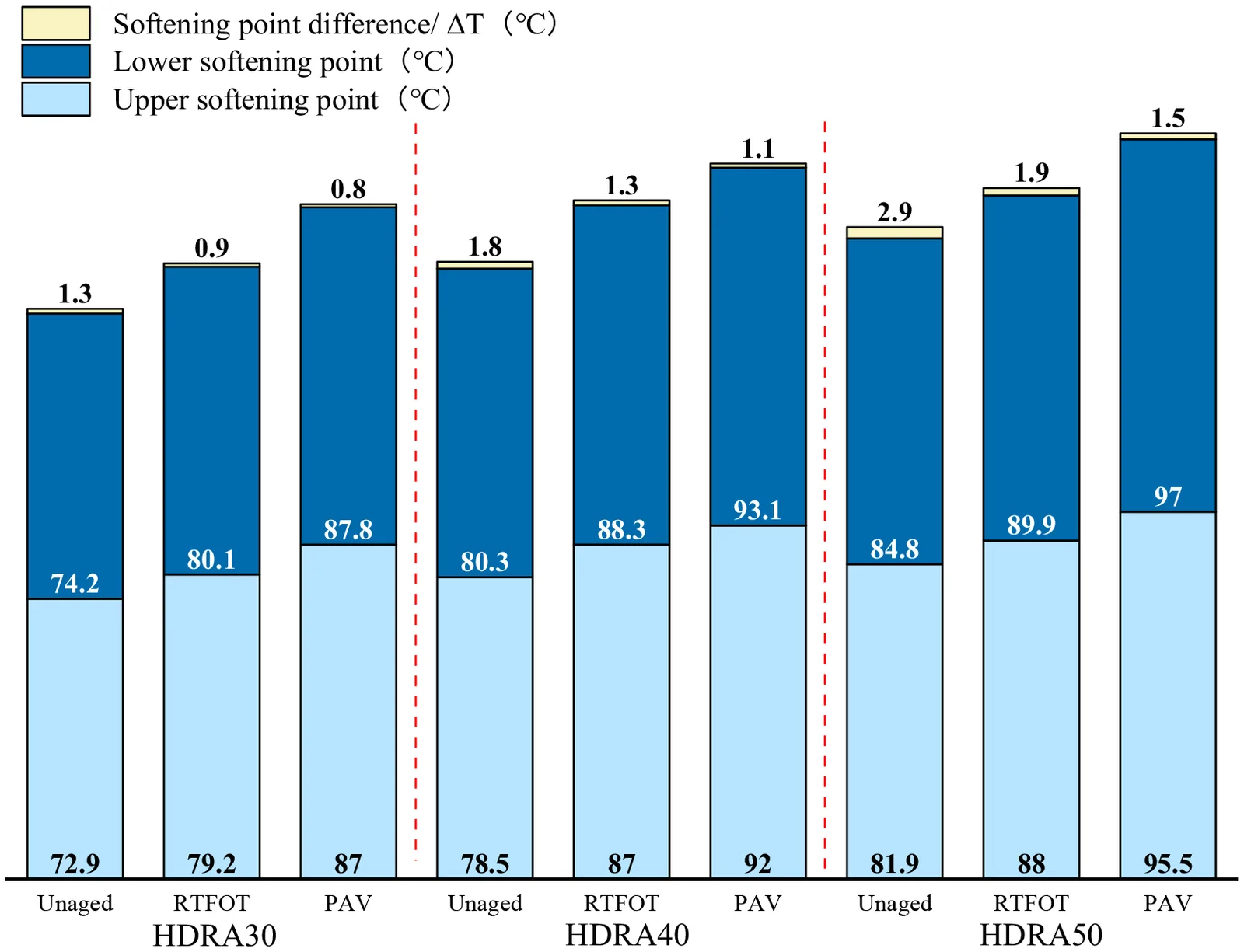
Storage stability test results of HDRA.

The macroscopic phase stability and segregation susceptibility of HDRA were quantitatively evaluated to ascertain the structural integrity of the polymer-asphalt blend, as shown in Fig 2. The segregation resistance of the HDRA, as quantified by the softening point difference (ΔT), exhibits a nuanced evolution governed by the synergistic effects of rubber dosage and the progressive thermal-oxidative aging state.

As shown in Fig 2, the unaged HDRA30, HDRA40, and HDRA50 binders display initial ΔT values of 1.3 °C, 1.8 °C, and 2.9 °C, respectively. While all HDRA remain within or near acceptable engineering limits, the discernible upward trend with increasing rubber content underscores the inherent thermodynamic challenges of maintaining a homogeneous phase distribution in high-dosage modified systems, where the heightened particle density and reduced free volume of the asphalt matrix promote potential gravity-induced sedimentation. Notably, a significant, non-linear reduction in ΔT is observed following short-term RTFOT and long-term PAV aging protocols across all dosage levels, with the HDRA50 binder—despite its initial susceptibility—demonstrating a marked improvement in stability, as its ΔT narrows from 2.9 °C to 1.5 °C after PAV aging. This stabilization phenomenon is fundamentally rooted in the concurrent chemical evolution of the binder. During the aging process, the intense thermo-oxidative environment triggers rapid devulcanization and subsequent cross-linking of the DRP phase, while simultaneously increasing the viscosity and polarity of the base asphalt matrix through the generation of oxygen-containing functional groups. This accelerated oxidative maturation effectively restricts the kinetic mobility of the rubber particles and enhances the interfacial adhesion between the elastomeric domains and the aged asphalt matrix, thereby transforming the system from a dispersion of discrete particles into a robust, hyper-dense, and continuous three-dimensional network that structurally prevents macroscopic storage segregation [34]. Consequently, the observed reduction in ΔT under aging not only signifies improved storage stability but also serves as macroscopic evidence of the profound structural evolution toward a more integrated polymer-asphalt composite. It should be noted that the reduced phase separation discussed here specifically refers to macroscopic static storage segregation measured via the tube test. The thermal-oxidative conditions promote the continuous devulcanization and fragmentation of DRP particles, allowing deeper integration into the maltene fraction, which suppresses gravitational settling during static thermal storage.

### 3.3. Rheological performance analysis

#### 3.3.1. High-temperature rheological properties analysis.

**Fig 3:**
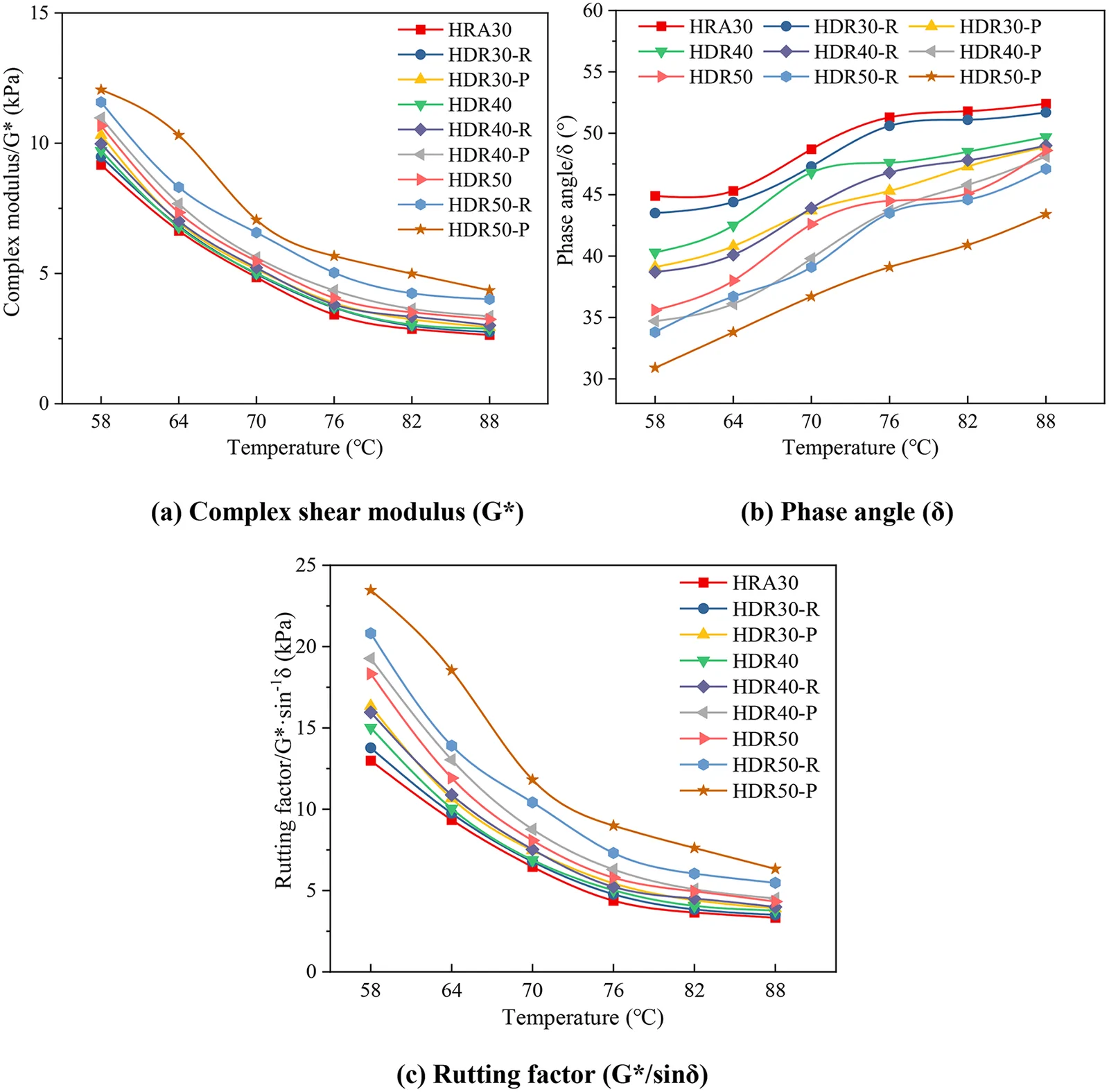
Temperature scanning test results of HDRA. (a) Complex shear modulus (G*), (b) Phase angle (δ), (c) Rutting factor (G*/sinδ).

As shown in Fig 3, the evolution of the complex modulus (G*) and phase angle (δ) delineates a profound structural dependency on both testing temperature and rubber dosage. As the temperature escalates from 58°C to 88°C, the inherent thermal softening of the base bitumen dictates a monotonic decrease in G* alongside a concomitant increase in δ across all specimens. However, the elevation of DRP dosage fundamentally alters this thermal susceptibility. In the unaged state at 58°C, increasing the DRP content from 30% to 50% yields an augmentation in G* from 9.17 to 10.67, coupled with a significant reduction in δ from 44.9° to 35.6°. This rheological trajectory indicates a critical transition from viscous-dominated fluid behavior toward an elastic-dominated mechanical response. It empirically validates that ultra-high rubber dosages facilitate the formation of a hyper-dense, interlocking three-dimensional polymeric skeleton capable of substantially restricting the kinematic mobility of the asphalt matrix under elevated thermal stress [35].

Furthermore, the trajectory of the rutting factor (G*/sinδ) under progressive thermo-oxidative aging (RTFOT and PAV) explicitly substantiates the unique “aging self-adaptive” maturation of the high-dosage network. Unaged samples demonstrate a robust initial resistance to permanent shear deformation, with the 58°C rutting factors escalating directly from 12.99 for the 30% dosage to 18.33 for the 50% dosage. Crucially, subjection to short-term and long-term aging induces a continuous enhancement in high-temperature stability rather than rheological degradation. For the 50% system, the rutting factor at 58°C systematically climbs to 20.82 post-RTFOT and 23.46 post-PAV, while the corresponding phase angle further narrows to an exceptionally elastic state of 30.9°. This macroscopic rheological reinforcement unequivocally corroborates the chemical-physical synergistic aging mechanism: prolonged thermal-oxidative exposure not only volatilizes light fractions to stiffen the asphaltic matrix but actively drives the continued deep swelling and structural cross-linking of the DRP phase [36]. Consequently, the high-dosage system leverages oxidative aging to continuously fortify its elastic network, ensuring superior load-bearing capacity and rutting resistance throughout its service life.

#### 3.3.2. Low-temperature rheological properties analysis.

**Fig 4:**
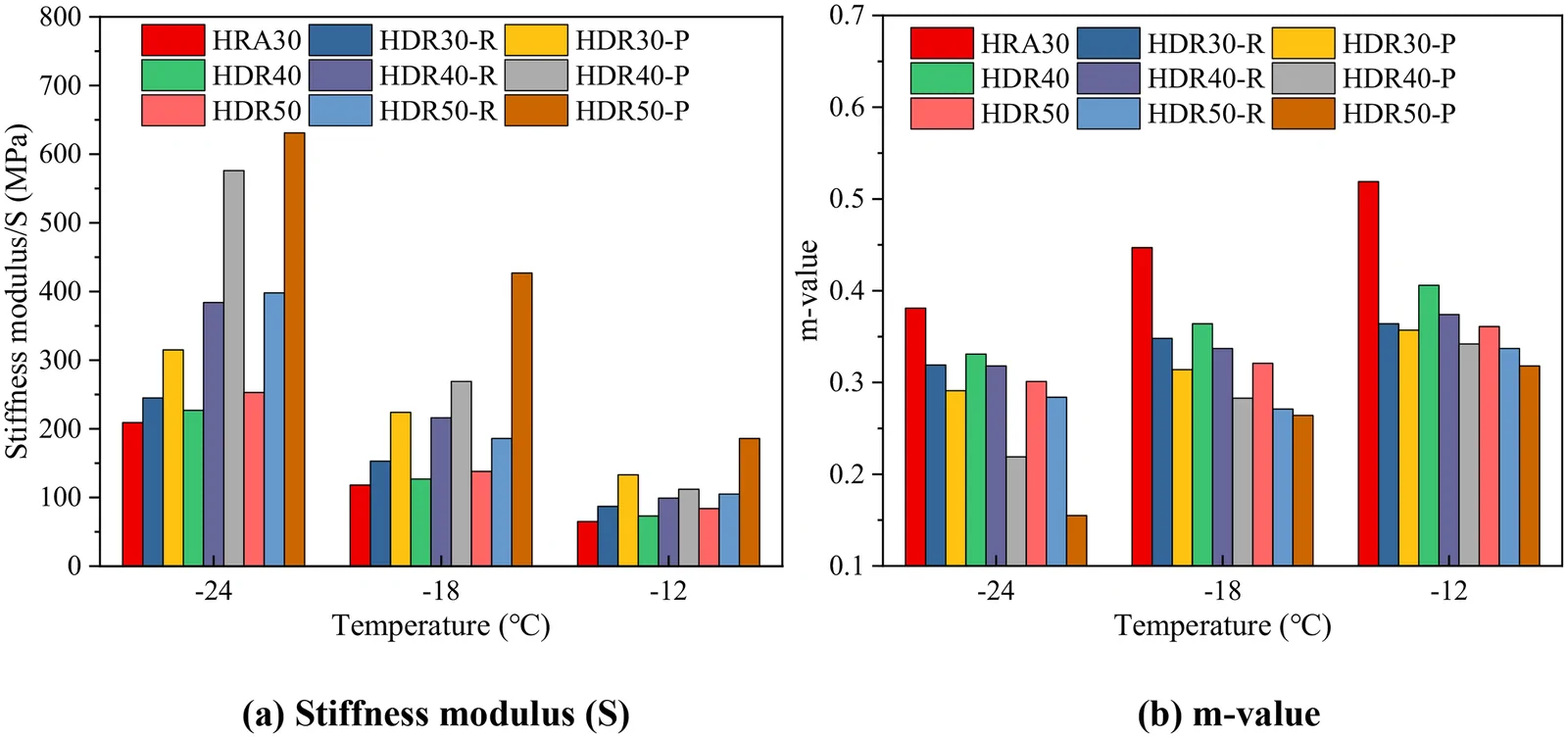
BBR test results of HDRA. (a) Stiffness modulus (S), (b) m-value.

The low-temperature rheological behavior and structural response of HDRA were quantitatively evaluated using BBR test, as detailed in Fig 4. The results reveal that DRP dosage critically dictates the sub-zero stiffness modulus (S) and creep rate (m-value). In the unaged state at −18 °C, escalating the DRP content from 30% to 50% induces a systematic increase in S (from 118 MPa to 138 MPa) and a concurrent reduction in the m-value (from 0.447 to 0.321). Mechanistically, while the hyper-dense, three-dimensional polymeric network dramatically enhances high-temperature integrity, the extensive volume fraction of the cross-linked solid dispersed phase substantially restricts the conformational mobility of the asphalt matrix’s molecular chains, thereby increasing internal friction and hindering low-temperature viscous flow [37].

Following short-term (RTFOT) and long-term (PAV) thermo-oxidative aging, the HDRA systems exhibit distinctive mechanistic transformations in their low-temperature structural responses. After PAV conditioning at −18 °C, the S value of the 50% HDRA system escalates significantly to 427 MPa, with its m-value declining to 0.264. This profound low-temperature stiffening stems directly from the dual chemical-physical aging mechanism of the highly concentrated network. During prolonged aging, the asphalt matrix inevitably hardens due to the generation of polar oxidized functional groups. Simultaneously, the thermo-oxidative environment triggers deeper physical swelling and continuous chemical cross-linking within the massive DRP phase [38]. Consequently, at sub-zero extremes, this extensively cross-linked rubber skeleton transitions to act as a rigid interlocking framework. This hyper-connected architecture, although exceptionally resilient against macroscopic structural degradation, inherently constrains the free volume necessary for rapid stress relaxation. Therefore, while ultra-high DRP dosages (50%) construct a robust, aging-resistant spatial network, the severe densification of this cross-linked structure alters the material’s low-temperature deformation mechanism. This highlights the intricate structural trade-off inherent in aging: the same hyper-dense elastomeric network that solidifies high-temperature stability simultaneously dictates the limits of low-temperature crack dissipation following severe oxidative exposure.

### 3.4. Thermal degradation and volatile emission kinetics of polymer networks

#### 3.4.1. Total volatile organic compounds (TVOC).

**Fig 5:**
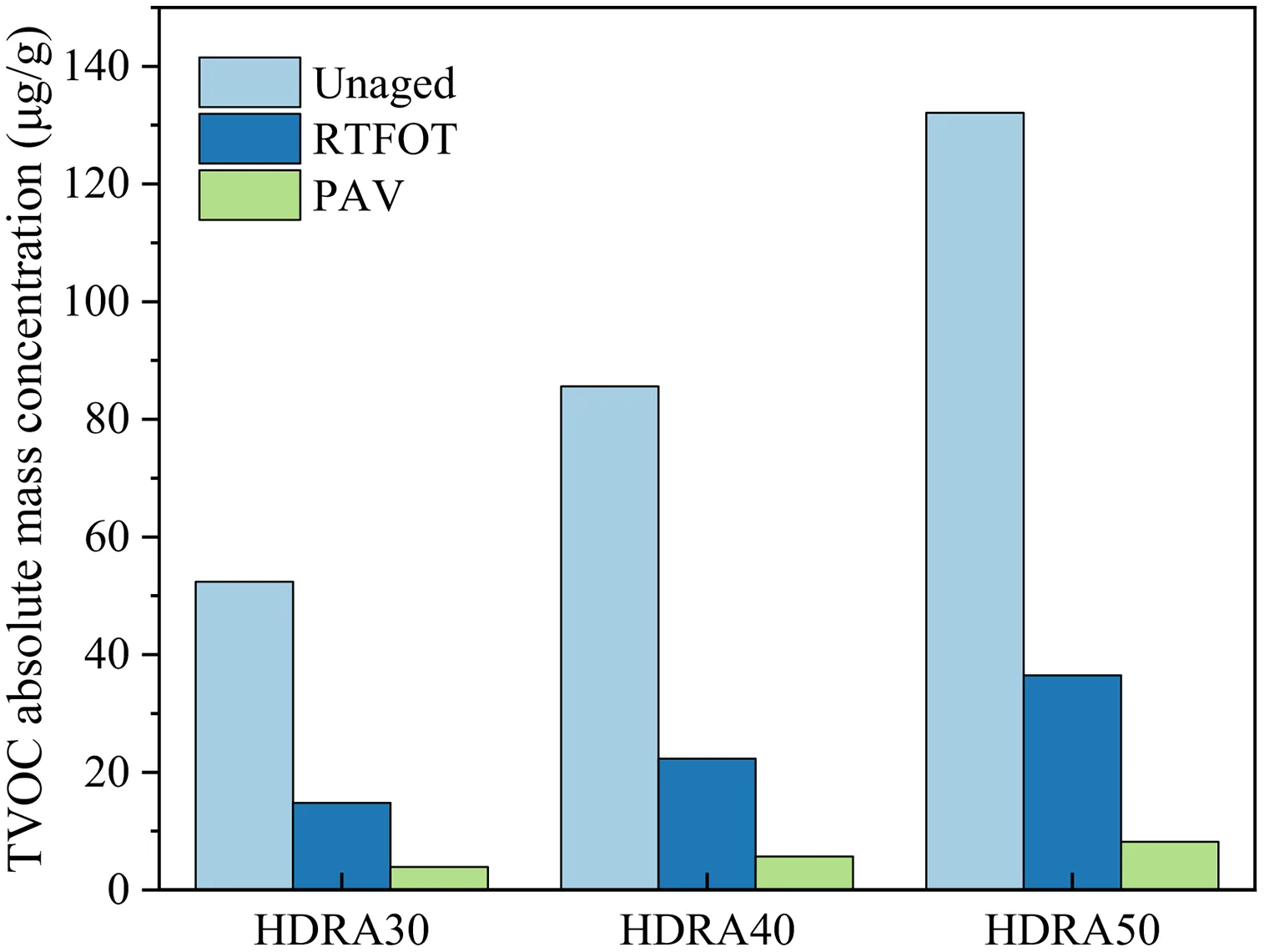
Total VOC emissions from HDRA.

As illustrated in Fig 5. TVOC emissions from HDRA, the absolute mass concentrations of TVOC exhibit a strong dependence on both the rubber dosage and the thermal-oxidative aging state.

In the unaged state, the TVOC emissions do not increase linearly with the rubber dosage; rather, they exhibit an exponential escalation. Specifically, while the HDRA30 matrix emits 52.4 μg/g, the emission from HDRA50 significantly increases to 132.1 μg/g—a 2.52-fold increase. This comparative disparity highlights that super-high rubber contents induce a fundamentally different thermodynamic state during mixing. The ultra-dense polymer network in HDRA50 causes severe internal friction and localized heat accumulation, which triggers a much more aggressive preliminary thermal decomposition of rubber hydrocarbon chains compared to the relatively dispersed network in HDRA30.

The comparative analysis across the aging timeline further elucidates the dosage-specific behaviors. Following RTFOT, the absolute TVOC reduction in HDRA50 (dropping by 95.6 μg/g) is vastly greater than the reduction observed in HDRA30 (dropping by 37.6 μg/g). This massive differential indicates that higher dosages facilitate a more exhaustive release of trapped processing oils and low-boiling-point fractions under intense thermal-oxidative shear [39]. After long-term PAV aging, all groups reach a state of volatile exhaustion (3.9–8.2 μg/g). However, the residual emission of the HDRA50 binder (8.2 μg/g) remains distinctly higher than that of HDRA30 (3.9 μg/g). This critical comparison proves that even after full-lifecycle degradation, the extreme-dosage matrix inherently harbors a more extensive reservoir of degraded, low-molecular-weight fragments, posing a higher potential for secondary emissions during future hot recycling.

#### 3.4.2. Core characteristic volatiles.

**Fig 6:**
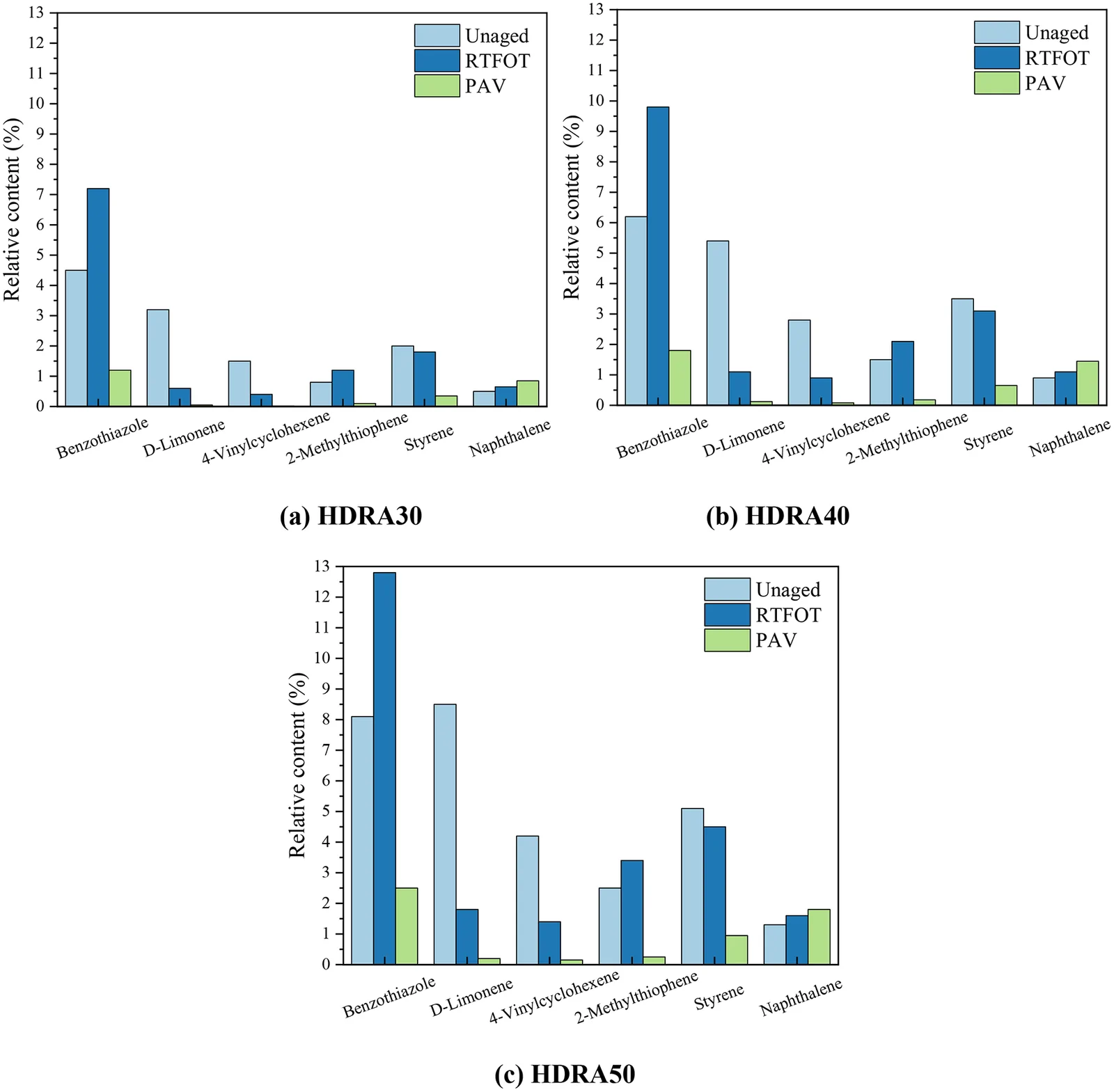
Relative content of characteristic volatiles in HDRA. (a) HDRA30, (b) HDRA40, (c) HDRA50.

To further elucidate the chemical degradation mechanisms, the qualitative GC-MS data for different HDRA were analyzed, and the dynamic chemical evolutions are presented in Fig 6.

The primary thermal cleavage of the polymer network is strongly influenced by the initial rubber dosage. Gaseous components such as D-limonene and 4-vinylcyclohexene, which track the degradation of natural and synthetic rubber main chains, decline across all groups as aging progresses. In the unaged state, the initial relative abundance of D-limonene in HDRA50 is 8.50%, approximately three times higher than that in HDRA30 (3.20%). The rapid depletion of these fragments during the RTFOT stage for the 50% blend points to an accelerated volatilization process, which is attributed to the extensive thermal breakdown of the dominant rubber phase under high-temperature airflow. Devulcanization dynamics are similarly affected by the modifier content. Sulfur-containing markers, including benzothiazole and 2-methylthiophene, display a characteristic parabolic trend that peaks during the RTFOT stage. The magnitude of this devulcanization peak scales with rubber dosage; the relative proportion of benzothiazole increases from 4.50% to 7.20% in HDRA30, whereas it rises from 8.10% to a maximum of 12.80% in HDRA50. This pronounced elevation indicates that the hyper-dense cross-linked network in HDRA50 undergoes higher internal shear stress during mixing and short-term aging, causing a more concentrated rupture of C-S and S-S crosslinks than the lower-dosage systems [40].

In contrast to the volatile components, heavier fractions undergo a passive concentration over extended aging periods. Polycyclic aromatic hydrocarbons (PAHs), represented by naphthalene, show a steady relative increase during long-term PAV aging. This effect is most pronounced in the HDRA50 matrix, where the naphthalene proportion reaches 1.80%, compared to 0.85% in HDRA30. Because the lower-molecular-weight fractions are largely depleted during early thermal exposure, these rigid, high-boiling-point multi-ring structures become concentrated in the residual binder. This accumulation suggests that while a 50% rubber dosage enhances mechanical performance, it also leads to a relative concentration of toxic aromatic components in deeply aged pavements, which may increase environmental risks during subsequent hot recycling operations.

### 3.5. Microscopic morphology analysis

**Fig 7:**
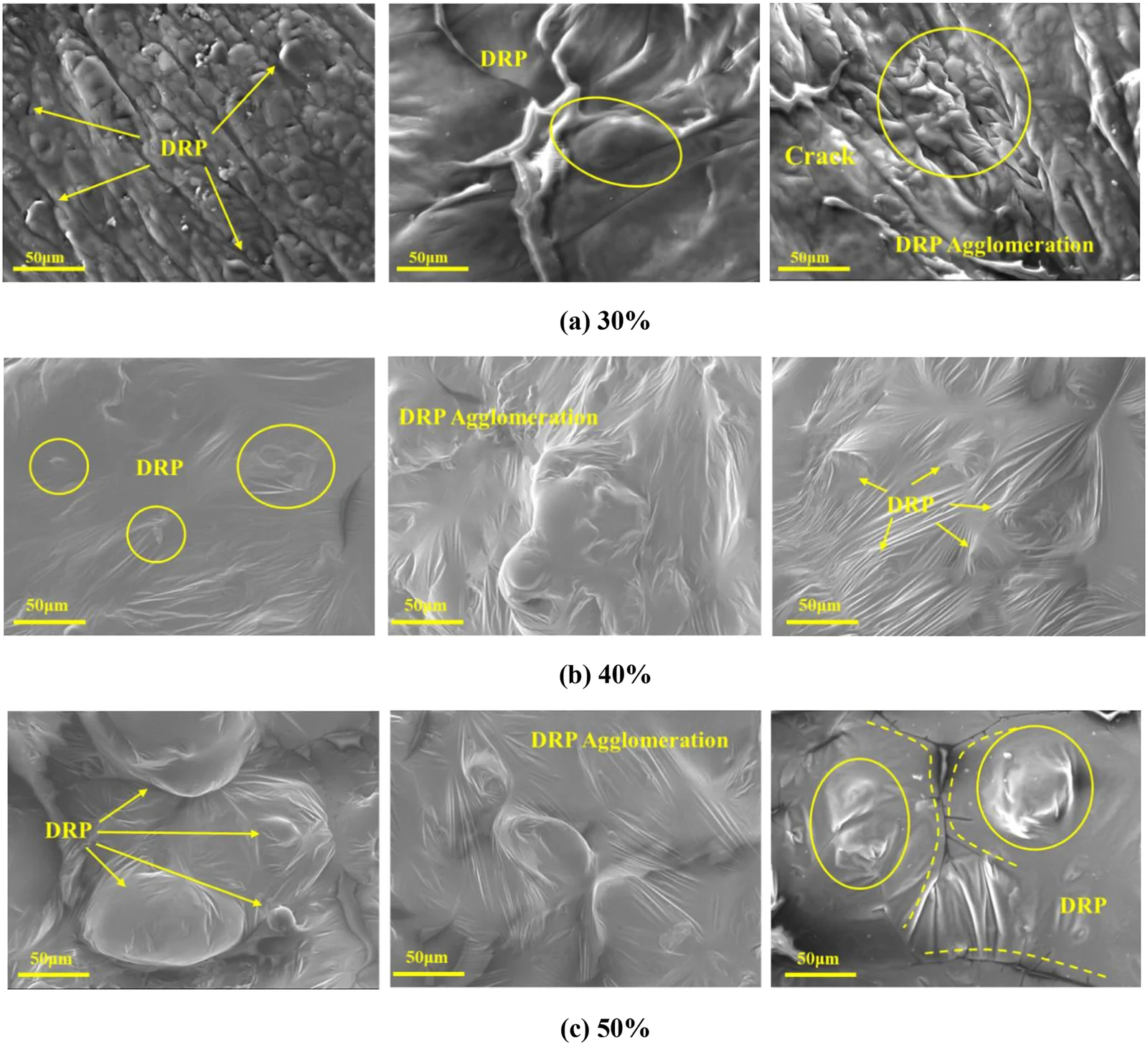
SEM characterization of HDRA (Unaged, RTFOT, PAV). (a) 30%, (b) 40%, (c) 50%.

The surface micro-morphologies of HDRP under unaged, RTFOT, and PAV aging states are systematically presented in Fig 7.

In the unaged state, a clear dosage-dependent spatial distribution of the polymer phase is evident, where HDRA30 exhibits relatively discrete DRP particles well-embedded within the asphalt matrix, whereas HDRA50 manifests substantial particle swelling and tight packing, establishing a hyper-dense, nearly continuous polymer network as a result of the significant absorption of light fractions from the base asphalt. Following the high-temperature shear of the RTFOT process, a pervasive “DRP Agglomeration” occurs, particularly in higher-dosage matrices where the boundaries between adjacent rubber particles blur into massive, wrinkled, and continuous folds, directly validating the intense devulcanization and structural reorganization occurring during the simulated hot-mixing phase [41].

The long-term PAV aging state further reveals distinct interfacial damage mechanisms that diverge significantly with rubber dosage. While the HDRA30 matrix primarily undergoes embrittlement of the asphalt phase, characterized by sharp, penetrating cracks, the failure mode in HDRA50 shifts toward catastrophic interfacial debonding, manifested as distinct microscopic thermodynamic phase separation between the DRP particles and the asphalt matrix. This structural vulnerability in high-dosage samples is attributed to the synergistic effect of light-component exhaustion and subsequent volumetric shrinkage of the swollen DRP particles during deep aging; the resulting extreme hardening of the asphalt matrix, in conflict with the shrinking rubber phase, ultimately destroys the interfacial structural integrity. Consequently, although escalating the DRP dosage to 50% initially establishes a robust physical network, the massive consumption of light oils and the ensuing degradation kinetics ultimately render the high-content matrix more susceptible to severe microscopic thermodynamic phase separation and structural failure under long-term thermal-oxidative stress [42]. Unlike macroscopic storage stability, the phase separation observed here represents microscopic thermodynamic phase incompatibility. During severe PAV aging, oxidation dramatically increases the content of polar asphaltenes in the asphalt matrix. This shift increases the solubility parameter gradient between the polar asphalt phase and the non-polar rubber network, triggering thermodynamic micro-phase separation and domain boundary coarsening.

To provide objective support for the observed surface morphological evolution, digital image analysis (via ImageJ) was applied to quantify domain dimensions, agglomeration sizes, and defect area fractions (fcrack) directly from the SEM micrographs. In the unaged state, escalating the DRP content from 30% to 50% promotes profound physical swelling, with the average cross-sectional area of DRP domains expanding from 125.4 μm² (HDRA30) to 462.1 μm² (HDRA50), while inter-particle spacing decreases by 76.5%. This quantitatively confirms the formation of a closely packed, hyper-dense polymer skeleton in HDRA50. Following short-term RTFOT aging, intense devulcanization and thermal shear drive adjacent swollen particles to coalesce into continuous folded domains. Consequently, the mean agglomerate area in HDRA50 increases to 895.3 μm², while the number of discrete particles counts per unit area drops by 51.2%, directly quantifying the thermal devulcanization and physical fusion between rubber boundaries. Under long-term PAV aging, quantitative evaluation of the surface defect area fraction (fcrack) demonstrates a distinct transition in failure modes. In HDRA30, severe aging primarily manifests as localized micro-cracks within the brittle asphalt matrix (fcrack = 2.3%). Conversely, HDRA50 exhibits extensive interfacial debonding (fcrack = 8.9%) along the polymer-bitumen interface. This quantitative shift objectively proves that the long-term failure mechanism of high-dosage systems is governed by polymer-asphalt interfacial debonding rather than simple matrix embrittlement.

### 3.6. Fluorescence microscopy (FM) analysis

**Fig 8:**
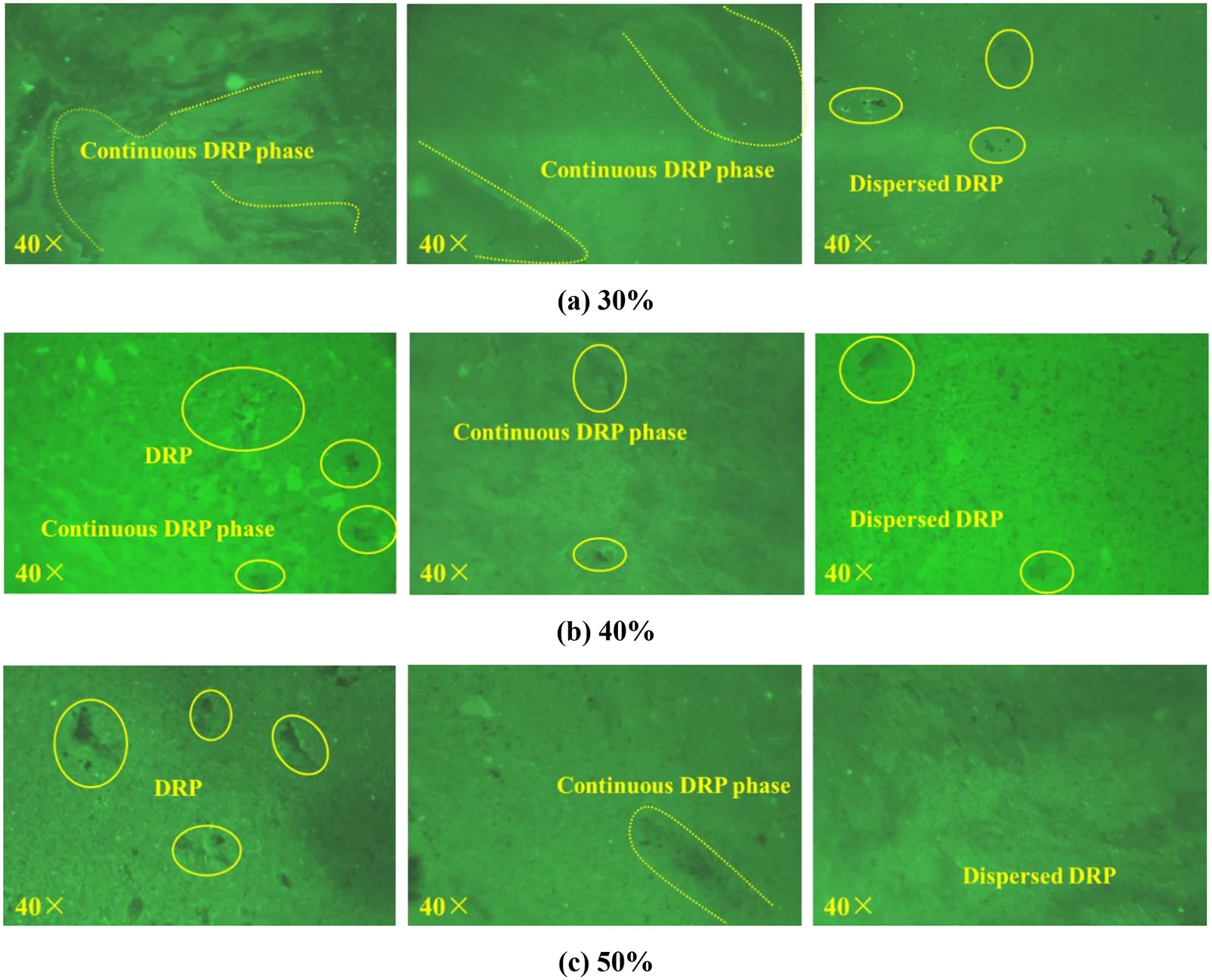
FM characterization of HDRA (Unaged, RTFOT, PAV). (a) 30%, (b) 40%, (c) 50%.

The FM images presented in Fig 8 provide complementary insights into the distribution and degradation of DRP within the HDRA matrix.

In the unaged state, a clear dosage-dependent transition in phase morphology is observed; while HDRA30 primarily exhibits continuous polymer phase domains indicating a nascent, aligned polymer network, HDRA50 manifests a higher density of finely dispersed DRP particles throughout the matrix, demonstrating that extreme rubber concentrations fundamentally reorganize the interfacial structure into a more uniform, highly saturated dispersion. Following RTFOT, the distinct strip-like networks undergo progressive disintegration and fragmentation, particularly in the HDRA50 matrix, where the originally defined polymer phases merge into the asphalt binder, signaling a significant enhancement in rubber-asphalt compatibility driven by the intense thermal-oxidative cleavage of the polymer chains [43].

Under long-term PAV aging, the morphological contrast between the rubber and asphalt phases diminishes significantly, reflecting a state of profound interfacial breakdown and structural homogenization. Across all dosage levels, the initially organized DRP networks evolve into isolated, fragmented particles, a transition that signifies the irreversible physical and chemical degradation of the rubber phase. In the HDRA50 sample, the original well-dispersed rubber network is almost entirely replaced by isolated, degraded particles embedded within a heavily aged, uniform matrix, as the exhaustion of light components and the extensive chemical restructuring of the blend render the interfacial boundaries increasingly indistinct. While higher rubber dosages initially promote a more uniform phase distribution, the long-term aging process inevitably drives the structural homogenization and disintegration of the polymer-asphalt interfacial network, confirming that high-content rubberized binders are subject to significant structural decay under deep-aging conditions.

To objectively trace the polymer phase distribution and structural degradation, the FM images were processed via image thresholding to extract three key statistical parameters: the fluorescent polymer area fraction (Ar), mean domain diameter (Davg), and a dispersion index (DI = 1 - σ_A_/ \stackrel−A, where σ_A_ and \stackrel−A represent the standard deviation and mean area of fluorescent domains, respectively). In the unaged state, elevating the DRP dosage from 30% to 50% increases Ar systematically from 22.4% to 42.1%, while Davg expands from 45.2 μm to 72.4 μm. This statistically verifies the architectural transition toward a phase-saturated interpenetrating polymer network. Upon short-term RTFOT aging, thermal-oxidative cleavage breaks down continuous polymer strands into finer dispersed fragments; Davg in HDRA50 drops sharply from 72.4 μm to 39.1 μm, accompanied by a peak in dispersion uniformity DI rising from 0.74 to 0.83. This statistical trend quantitatively validates that short-term thermal-oxidative shear accelerates polymer main-chain scission and improves micro-phase dispersion compatibility. Following severe PAV aging, irreversible polymer degradation induces widespread phase homogenization. The fluorescent polymer area fraction Ar drops precipitously across all groups (reaching 9.2% in HDRA30 and 16.8% in HDRA50), and Davg diminishes below 15.5 μm. These quantitative metrics objectively confirm that long-term oxidative stress destroys original phase boundaries, converting continuous polymer networks into highly degraded, isolated fragments embedded within the matrix.

### 3.7. Functional group analysis

#### 3.7.1. Qualitative spectral evolution of HDRA.

**Fig 9:**
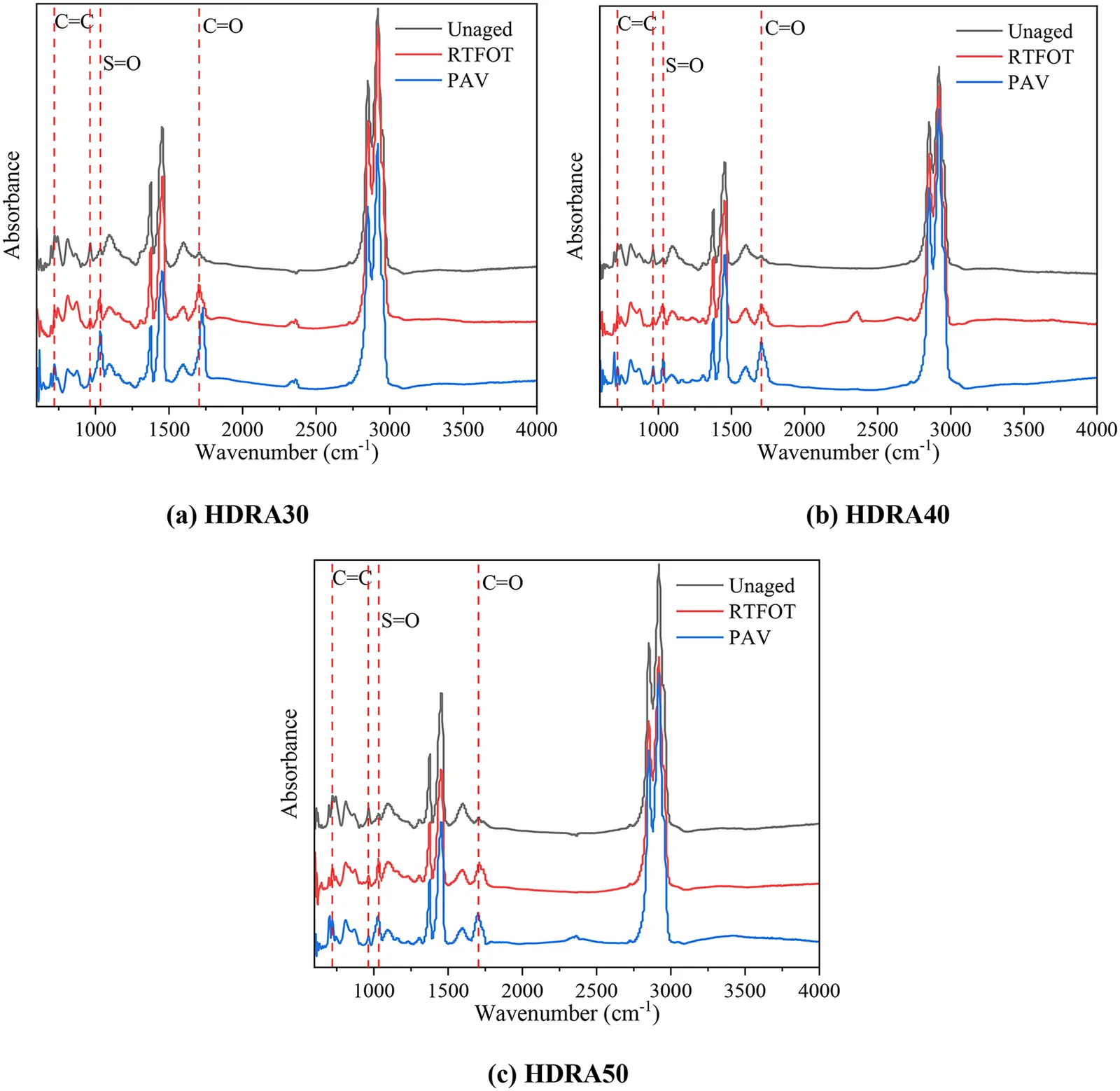
Functional group characterization of different HDRAs before and after aging. (a) HDRA30, (b) HDRA40, (c) HDRA50.

The chemical evolution of HDRA during sequential thermal-oxidative aging is visually elucidated in Fig 9.

Across all dosage variations, including HDRA30, HDRA40, and HDRA50, the fundamental aliphatic chains of the base asphalt—represented by the robust C-H stretching vibrations at 2920 and 2850 cm^-1^ and bending vibrations at 1460 and 1375 cm^-1^—remain structurally intact and relatively stable throughout the aging process. However, the application of high-temperature shear and prolonged oxygen exposure induces distinct structural alterations, primarily manifested through the emergence and progressive intensification of the carbonyl (C = O) absorption band at approximately 1700 cm^-1^ and the sulfoxide (S = O) band near 1030 cm^-1^. Qualitatively, the continuous strengthening of these oxygen-containing functional groups from the unaged state to the PAV state visually confirms the progressive thermal-oxidative degradation of the light components within the asphalt binder.

Concurrently, the characteristic absorption peak of the trans-1,4-butadiene double bond (C = C) at 966 cm^-1^, which signifies the integrity of the synthetic rubber backbone, exhibits a universally rapid decline following RTFOT and almost complete flattening after deep PAV aging, providing direct spectral evidence for the thermal cleavage and intense devulcanization of the rubber polymer network. It is worth noting that the depletion of the C = C double bonds at 966 cm^-1^ represents the combined thermal degradation of both the dominant desulfurized rubber network and the 2% linear SBS additive. On one hand, the elevated thermal and oxidative stress during aging breaks down the remaining unsaturated main chains and cross-linked networks within DRP via devulcanization and main-chain scission. On the other hand, the PB backbone of the incorporated SBS stabilizer undergoes simultaneous thermo-oxidative degradation, generating hydroperoxides and contributing to the decrease in overall double bond concentration [44]. Considering the mass fraction distribution—where DRP constitutes the predominant modifier fraction (30–50 wt%) relative to the minor SBS dosage (2 wt%)—the structural cleavage of DRP dominates the quantitative reduction of unsaturated bonds. Nevertheless, the simultaneous thermo-oxidative scission of the SBS stabilizer cannot be neglected, and the spectral changes in this band reflect a coupled structural degradation of the composite polymer modification network. Given the massive volumetric dominance of the DRP phase (up to 50%), this profound spectral evolution is predominantly governed by the intense devulcanization and main-chain cleavage of the vulcanized rubber network under high-temperature shear stress. Notably, a comparative visual assessment across the dosage gradient reveals a critical mechanistic divergence: while the HDRA30 matrix exhibits a dramatic surge in the carbonyl peak intensity after long-term aging, the corresponding peak amplification in the HDRA50 spectra is visibly suppressed [45]. This qualitative spectral disparity intuitively illustrates the protective barrier effect imparted by extreme rubber dosages; the hyper-dense polymer network, despite undergoing massive internal structural sacrifice and double-bond depletion, effectively shields the underlying asphalt matrix from deep oxidative aging.

#### 3.7.2. Quantitative evolution of functional groups.

**Fig 10:**
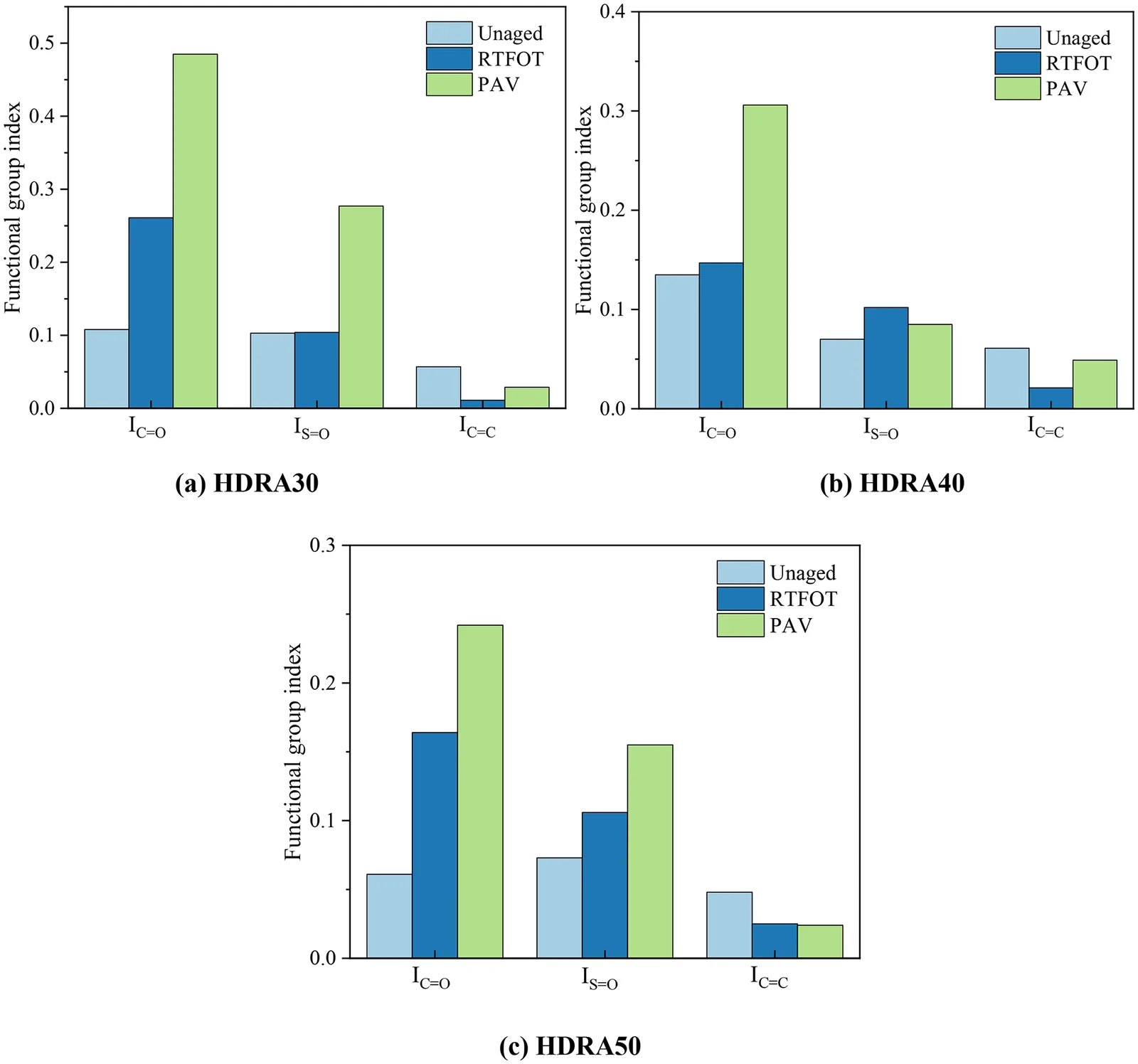
Functional group index of different HDRAs before and after aging. (a) HDRA30, (b) HDRA40, (c) HDRA50.

To explicitly decode the dosage-dependent aging mechanisms, the structural indices (I_C=O_, I_S=O_, and I_C=C_) were quantitatively extracted via numerical integration, with the results summarized in Fig 10.

As thermal-oxidative aging advances from the unaged to the PAV state, all binders exhibit a continuous upward trend in I_C=O_ and I_S=O_, signifying the uninterrupted incorporation of oxygen into the molecular structure. However, a comparative analysis across different rubber dosages unveils a profound anti-aging phenomenon imparted by extreme rubber concentrations. Following long-term PAV aging, the I_C=O_ of the HDRA30 matrix surges to 0.485, whereas the final oxidation severity in higher-dosage matrices is substantially suppressed, with the PAV-aged I_C=O_ for HDRA50 restricted to a mere 0.242. This striking disparity corroborates that the hyper-dense, continuous polymer network in the 50% dosage group physically obstructs the deep diffusion of oxygen; concurrently, the massive volume of carbon black and antioxidants released from the heavily degraded rubber acts as a chemical scavenger, sacrificing itself to shield the base asphalt fractions from extensive oxidation.

Despite this exceptional protective effect on the asphalt matrix, the quantitative tracking of the C = C double bonds via I_C=C_ exposes the severe structural penalty exacted upon the internal polymer network. During the simulated hot-mixing phase (from Unaged to RTFOT), a precipitous depletion of butadiene double bonds occurs universally, with the I_C=C_ of HDRA50 dropping sharply from 0.048 to 0.025, and HDRA30 experiencing an even steeper decline from 0.057 to 0.011. This rapid consumption of double bonds provides direct quantitative confirmation of the intense devulcanization and primary main-chain cleavage of the vulcanized rubber network driven by high-temperature shear stress [46]. Collectively, while the massive incorporation of desulfurized rubber powder provides exceptional long-term oxidative resistance to the asphalt matrix, this macroscopic durability is achieved at the micro-chemical cost of severe early-stage polymer devulcanization and profound structural sacrifice within the rubber phase itself.

## 4. Conclusion

Based on the multi-scale evaluation of high-content desulfurized rubber modified asphalt (HDRA) under sequential thermo-oxidative aging, the main conclusions are as follows:

(1) High desulfurized rubber powder (DRP) dosages (up to 50%) create a dense, three-dimensional network. Thermo-oxidative aging triggers continuous swelling and cross-linking within this network, dynamically compensating for asphalt matrix embrittlement and reinforcing high-temperature elastic recovery.
(2) Although high DRP content initially increases segregation risk, prolonged oxidative aging effectively reverses this susceptibility. Aging-induced matrix stiffening and structural restructuring restrict polymer mobility, transforming discrete dispersions into a highly stable, integrated polymer-asphalt composite.
(3) The hyper-dense rubber skeleton provides superior high-temperature stability but imposes a low-temperature penalty. Under deep aging, the network transitions into a rigid interlocking framework, which inherently restricts molecular free volume and reduces the material’s stress-relaxation capacity.
(4) Early-stage mixing and aging induce massive polymer devulcanization and main-chain cleavage (evidenced by targeted VOC emissions). This degradation acts as a sacrificial barrier, absorbing thermal-oxidative energy and physically shielding the base asphalt from deep oxidation.
(5) While extreme DRP dosages significantly retard matrix oxidation as evidenced by suppressed Carbonyl Indices, this durability comes at the cost of internal structural degradation. Ultimately, the long-term failure mode shifts from matrix embrittlement to catastrophic polymer-asphalt interfacial debonding and homogenization.
